# Supplementary material for: Revealing broken valley symmetry of quantum emitters in WSe2 with chiral nanocavities
Source: Nat Commun. 2023 Jul 17;14:4265. doi: 10.1038/s41467-023-39972-7 (PMC10352360; doi:10.1038/s41467-023-39972-7)
Supplement: Supplementary file 1 — Supplementary Information [file 41467_2023_39972_MOESM1_ESM.pdf]

Supplementary Information for Revealing broken valley symmetry of quantum emitters in WSe_2 with chiral nanocavities

Longlong Yang,[1,2] Yu Yuan,[1,2] Bowen Fu,[3] Jingnan Yang,[3] Danjie Dai,[1,2] Shushu Shi,[1,2] Sai Yan,[1,2] Rui Zhu,[1,2] Xu Han,[4] Hancong Li,[3] Zhanchun Zuo,[1,2] Can Wang,[1,2,5,*] Yuan Huang,[4,†] Kuijuan Jin,[1,2,5] Qihuang Gong,[3,6] and Xiulai Xu[3,6,‡]

[1]*Beijing National Laboratory for Condensed Matter Physics, Institute of Physics, Chinese Academy of Sciences, Beijing 100190, China*
[2]*CAS Center for Excellence in Topological Quantum Computation and School of Physical Sciences, University of Chinese Academy of Sciences, Beijing 100049, China*
[3]*State Key Laboratory for Mesoscopic Physics and Frontiers Science Center for Nano-optoelectronics, School of Physics, Peking University, 100871 Beijing, China*
[4]*Advanced Research Institute of Multidisciplinary Science, Beijing Institute of Technology, Beijing 100081, China*
[5]*Songshan Lake Materials Laboratory, Dongguan, Guangdong 523808, China*
[6]*Peking University Yangtze Delta Institute of Optoelectronics, Nantong, Jiangsu 226010, China*

Contents:

Supplementary Note 1 - Dynamics for the interaction between intervalley defect exciton and chiral plasmon field.
Supplementary Note 2 - Resonance of chiral plasmon lattices.
Supplementary Note 3 - The *g*-factors of QEs in WSe_2 monolayer.
Supplementary Note 4 - Circular photoluminescence polarization of the emission from WSe_2 monolayer on chiral plasmon.
Supplementary Note 5 - The power dependent PL and linear polarization-dependent PL of defects from WSe_2 layers.
Supplementary Note 6 - Optical characterizations of the interaction between chiral plasmon and $MoSe_2$ monolayer.

* canwang@iphy.ac.cn
† yhuang@bit.edu.cn
‡ xlxu@pku.edu.cn

SUPPLEMENTARY NOTE 1 - DYNAMICS FOR THE INTERACTION BETWEEN INTERVALLEY DEFECT EXCITON AND CHIRAL PLASMON FIELD.

The time evolution of a quantum system interacting with its environment is governed by the Liouville-von Neumann equation,

$$\frac{d}{dt}\rho_T(t) = -i[H_T(t), \rho_T(t)] \tag{1}$$

which is the equivalent of the Schrödinger equation in the density matrix formalism. H_T here is the total Hamiltonian: $H_T = H_{sys} + H_{env} + H_{int}$, which includes the system Hamiltonian H_{sys}, the environment Hamiltonian H_{env} and the interaction Hamiltonian H_{int} between the system and its environment. Here, since we are more interested in the dynamics of the system than that of the environment, we can obtain the reduced density matrix of the system $\rho = Tr_{env}[\rho_T]$ by tracing over the environment degrees of freedom. Therefore, the aim is to obtain the differential equation that the reduced density matrix ρ satisfies. The most general equation of the evolution for reduced density matrix is the Lindblad master equation [1, 2]:

$$\dot{\rho} = -i[H, \rho] + \sum_n \hat{C}_n \rho \hat{C}_n^\dagger - \sum_n \frac{1}{2}(\hat{C}_n^\dagger \hat{C}_n \rho + \rho \hat{C}_n^\dagger \hat{C}_n) \equiv \mathcal{L}\rho \tag{2}$$

where the term $-i[H, \rho]$ describes the Hermitian part of the evolution of the system we are interested in. $\hat{C}_n = \sqrt{\gamma_n}\hat{A}_n$ are the collapse operators, and $\hat{A}_n$ are the operators associated with the coupling of the system to the environment, and γ_n are the corresponding dissipation rates. The items containing $\hat{C}_n$ describe the non-hermitian evolution of the system due to its coupling to the environment, and is responsible for irreversible dissipation. The master equation can be expressed by $\mathcal{L}$, so-called Liouvillian superoperator. For a given Liouvillian, we can find a steady-state solution for ρ that satisfies $\mathcal{L}\rho = 0$.

The level scheme for the QE in WSe_2 monolayer is shown in Fig. 1b in the main text. The dark exciton state $|c_{K'(K)}\rangle$ hybridizes with the lower localized defect state $|d_{\uparrow(\downarrow)}\rangle$. Then the transition $|d_{\uparrow(\downarrow)}\rangle \leftrightarrow |\upsilon_{K(K')}\rangle$ is coupled to the chiral plasmon mode $\hbar\omega_{pl}\hat{a}^\dagger_{\sigma^+}\hat{a}_{\sigma^+}$ and $\hbar\omega_{pl}\hat{a}^\dagger_{\sigma^-}\hat{a}_{\sigma^-}$ with coupling strength g_{σ^+} and g_{σ^-}, respectively. The quantized chiral plasmon mode with resonance energy $\hbar\omega_{pl}$ is described by circularly polarized operators $\hat{a}_{\sigma^\pm} = \frac{1}{\sqrt{2}}(\hat{a}_1 \pm i\hat{a}_2)$ satisfying $[\hat{a}_{\sigma^\pm}, \hat{a}^\dagger_{\sigma^\pm}] = 1$ [3, 4]. Here, we assume that the external pump laser brings the exciton states into the excited intervalley momentum-forbidden dark exciton states, where it decay nonradiatively with a hybridization rate γ_{hyb} to the optically active defect states $|d\rangle$. This process will help us to separate the excitation dynamics, that means the plasmon field will not interact with the excitation process [5]. For the radiative transition of a QE, the plasmon nanocavity will strongly modify the radiation dynamics. Thus, using rotating wave approximation, the coherent part of the QE-CPR dynamics is described by the Hamiltonian

$$H = H_{QE} + H_{plasmon} + H_{pump} + H_{int-\sigma^+} + H_{int-\sigma^-} \tag{3}$$

$$H_{QE} = -\hbar\omega_{cd}\,|d_{\uparrow(\downarrow)}\rangle\langle d_{\uparrow(\downarrow)}| - (\hbar\omega_{QE} + \hbar\omega_{cd})\,|\upsilon_{K(K')}\rangle\langle\upsilon_{K(K')}| \tag{4}$$

$$H_{plasmon} = \hbar\omega_{pl}\hat{a}^\dagger_{\sigma^+}\hat{a}_{\sigma^+} + \hbar\omega_{pl}\hat{a}^\dagger_{\sigma^-}\hat{a}_{\sigma^-} \tag{5}$$

$$H_{pump} = \Omega(e^{i\hbar\omega_L t}\,|\upsilon_{K(K')}\rangle\langle c_{K'(K)}| + e^{-i\hbar\omega_L t}\,|c_{K'(K)}\rangle\langle\upsilon_{K(K')}|) \tag{6}$$

$$H_{int-\sigma^+} = g_{\sigma^+}(|d_{\uparrow(\downarrow)}\rangle\langle\upsilon_{K(K')}|\,\hat{a}_{\sigma^+} + |\upsilon_{K(K')}\rangle\langle d_{\uparrow(\downarrow)}|\,\hat{a}^\dagger_{\sigma^+}) \tag{7}$$

$$H_{int-\sigma^-} = g_{\sigma^-}(|d_{\uparrow(\downarrow)}\rangle\langle\upsilon_{K(K')}|\,\hat{a}_{\sigma^-} + |\upsilon_{K(K')}\rangle\langle d_{\uparrow(\downarrow)}|\,\hat{a}^\dagger_{\sigma^-}) \tag{8}$$

In the rotating frame with laser frequency ω_L by utilizing the unitary transformation U,

$$U(t) = exp[i\frac{\hbar\omega_L}{2}(|c_{K'(K)}\rangle\langle c_{K'(K)}| + |d_{\uparrow(\downarrow)}\rangle\langle d_{\uparrow(\downarrow)}| - |\upsilon_{K(K')}\rangle\langle\upsilon_{K(K')}|)t - i\hbar\omega_L(\hat{a}^\dagger_{\sigma^+}\hat{a}_{\sigma^+} + \hat{a}^\dagger_{\sigma^-}\hat{a}_{\sigma^-})t] \tag{9}$$

the Hamiltonian will be time-independent and can be rewritten as

$$H^I = U^\dagger(t)HU(t) - iU^\dagger(t)\frac{\partial}{\partial t}U(t) \tag{10}$$

that is

$$H^I_{QE} = \frac{\hbar\omega_L}{2}\left|c_{K'(K)}\right\rangle\left\langle c_{K'(K)}\right| + (-\hbar\omega_{cd} + \frac{\hbar\omega_L}{2})\left|d_{\uparrow(\downarrow)}\right\rangle\left\langle d_{\uparrow(\downarrow)}\right| + (-\hbar\omega_{QE} - \hbar\omega_{cd} - \frac{\hbar\omega_L}{2})\left|\upsilon_{K(K')}\right\rangle\left\langle \upsilon_{K(K')}\right| \tag{11}$$

$$H^I_{plasmon} = (\hbar\omega_{pl} - \hbar\omega_L)(\hat{a}^\dagger_{\sigma^+}\hat{a}_{\sigma^+} + \hat{a}^\dagger_{\sigma^-}\hat{a}_{\sigma^-}) \tag{12}$$

$$H^I_{pump} = \Omega(\left|\upsilon_{K(K')}\right\rangle\left\langle c_{K'(K)}\right| + \left|c_{K'(K)}\right\rangle\left\langle \upsilon_{K(K')}\right|) \tag{13}$$

$$H^I_{int-\sigma^+} = g_{\sigma^+}(\left|d_{\uparrow(\downarrow)}\right\rangle\left\langle \upsilon_{K(K')}\right|\hat{a}_{\sigma^+} + \left|\upsilon_{K(K')}\right\rangle\left\langle d_{\uparrow(\downarrow)}\right|\hat{a}^\dagger_{\sigma^+}) \tag{14}$$

$$H^I_{int-\sigma^-} = g_{\sigma^-}(\left|d_{\uparrow(\downarrow)}\right\rangle\left\langle \upsilon_{K(K')}\right|\hat{a}_{\sigma^-} + \left|\upsilon_{K(K')}\right\rangle\left\langle d_{\uparrow(\downarrow)}\right|\hat{a}^\dagger_{\sigma^-}) \tag{15}$$

The collapse operators for the incoherent part are

$$\hat{C}_{QE} = \sqrt{\gamma_{QE}}\left|\upsilon_{K(K')}\right\rangle\left\langle d_{\uparrow(\downarrow)}\right|,\ \hat{C}_{hyb} = \sqrt{\gamma_{hyb}}\left|d_{\uparrow(\downarrow)}\right\rangle\left\langle c_{K'(K)}\right| \tag{16}$$

$$\hat{C}_{pl-\sigma^+} = \sqrt{\gamma_{pl}}\hat{a}_{\sigma^+},\ \hat{C}_{pl-\sigma^-} = \sqrt{\gamma_{pl}}\hat{a}_{\sigma^-} \tag{17}$$

Taking the above Hamiltonian and the collapse operators into the Lindblad master equation, we can find a steady-state solution for ρ satisfying $\mathcal{L}\rho = 0$ and calculate the photon outputs of coupled QE-CPR system using QuTip [6, 7]. For the specific parameters used for calculation, we take the energy of pump laser $\hbar\omega_{pl} = 2.33$ eV (532 nm laser), the low energy peak of QE under magnetic field $\hbar\omega_{QE} = E_0 - (\alpha + m)\mu_B B$ (see section 3 for details), the linewidth of QE $\gamma_{QE} = 180$ µeV (see Fig. 2g in the main text), energy difference between dark conduction band and defect level $\hbar\omega_{cd} = E_\Delta - \hbar\omega_{QE} - (2\alpha + m)\mu_B B$. The band gap at K ($K^{'}$) is taken as $E_{gap} = 2.0$ eV and the energy difference between defect level and conduction band caused by strain is taken as $E_s = 0.3$ eV [8], thus $E_\Delta = E_{gap} - E_s = 1.7$ eV. We assume that the rate of hybridization is comparable to the decay of intervalley dark exciton, which is on the order of several meV [9]. The plasmon mode is taken from L1 that gives energy $\hbar\omega_{pl} \approx 1.65$ eV and linewidth $\gamma_{pl} \approx 180$ meV, as shown in Fig. 2c in the main text. For the plasmon mode, its decay consists of two parts, one is the radiation into free space (γ^r_{pl}) and the other is the ohmic loss in the metal (γ^0_{pl}), with the total decay rate $\gamma_{pl} = \gamma^r_{pl} + \gamma^0_{pl}$ [10, 11]. At 4.2 K, the electric resistivity of gold drops by $\sim 10^{-2} - 10^{-3}$ times compared to that of room temperature [12]. Thus, the contribution of electron-phonon scattering to the damping of plasmon will be severely reduced due to the low temperature, suggesting the output power of γ^0_{pl} drops by the same order of magnitude, that is, $\gamma_{pl} \approx \gamma^r_{pl}$.

To obtain DCP_{QE}, we first calculate the outputs of coupled QE and circularly polarized plasmon, that is, $\Phi_{QE-pl_{\sigma\pm}} = \langle \hat{C}^\dagger_{QE-pl_{\sigma\pm}}\hat{C}_{QE-pl_{\sigma\pm}}\rangle = \langle(\sqrt{\gamma_{QE}}\left|\upsilon_{K(K')}\right\rangle\left\langle d_{\uparrow(\downarrow)}\right| + \sqrt{\gamma^r_{pl}}\hat{a}_{\sigma\pm})^\dagger(\sqrt{\gamma_{QE}}\left|\upsilon_{K(K')}\right\rangle\left\langle d_{\uparrow(\downarrow)}\right| + \sqrt{\gamma^r_{pl}}\hat{a}_{\sigma\pm})\rangle = \Phi_{QE} + \Phi_{pl_{\sigma\pm}} + \Phi_{cross}$, including output of quantum emitter Φ_{QE}, polarized plasmon $\Phi_{pl_{\sigma\pm}} = \langle \hat{C}^\dagger_{pl_{\sigma\pm}}\hat{C}_{pl_{\sigma\pm}}\rangle$ and the cross term Φ_{cross}. Since the last two terms are co-polarized, we can obtain the degree of circular polarization of this part of the coupled system, that is, $DCP_{QE-pl} = (\Phi_{QE-pl_{\sigma+}} - \Phi_{QE-pl_{\sigma-}})/(\Phi_{QE-pl_{\sigma+}} + \Phi_{QE-pl_{\sigma-}} - 2\Phi_{QE})$. Experimentally measured degree of circular polarization also contains the circularly polarized components of QE ($\Phi_{QE_{\sigma\pm}}$), so it can be written as,

$$DCP = \frac{(\Phi_{QE_{\sigma+}} + \Phi_{QE-pl_{\sigma+}} - \Phi_{QE}) - (\Phi_{QE_{\sigma-}} + \Phi_{QE-pl_{\sigma-}} - \Phi_{QE})}{\Phi_{QE_{\sigma+}} + \Phi_{QE-pl_{\sigma+}} + \Phi_{QE_{\sigma-}} + \Phi_{QE-pl_{\sigma-}} - 2\Phi_{QE}} \tag{18}$$

where $\Phi_{QE} = \Phi_{QE_{\sigma+}} + \Phi_{QE_{\sigma-}}$. By calculating the $\beta = (\Phi_{QE-pl_{\sigma+}} + \Phi_{QE-pl_{\sigma-}} - 2\Phi_{QE})/\Phi_{QE}$, we can obtain the degree of circular polarization of QE, that is, $DCP_{QE} = (\Phi_{QE_{\sigma+}} - \Phi_{QE_{\sigma-}})/(\Phi_{QE_{\sigma+}} + \Phi_{QE_{\sigma-}}) = DCP + \beta(DCP - DCP_{QE-pl})$, Figure S1 shows the power outputs of the coupled QE-CPR system with g_{σ^-} dominated.

For the QE in WSe_2, whether the transition of QE is dominated by the valley-dependent optical selection rules will lead to completely different experimental results. As shown in Fig. S2, the incomplete circular polarized emission can be caused by different mechanisms. If it is due to the valley polarization, then the decrease in DCP is caused

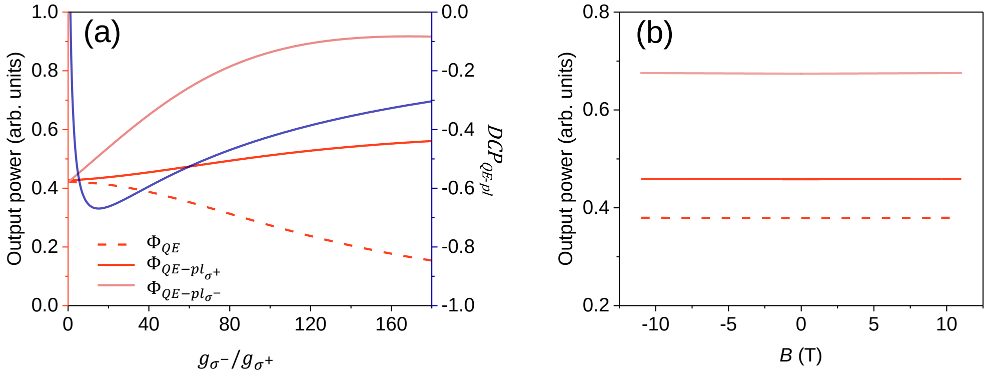

Supplementary Fig. 1. Power outputs of the coupled QE-CPR system with g_{σ^-} dominated. (a) Power outputs of different channels versus the chiral coupling strength ratio ($g_{\sigma^+} = 1$ meV). The coupling between QE and σ^- polarized plasmon field component is larger than that of σ^+ polarized due to $g_{\sigma^-} > g_{\sigma^+}$. (b) Power outputs as a function of B ($g_{\sigma^-} = \gamma_{pl}/4$). The radiation of the system is almost independent of the energy shift caused by the Zeeman splitting, indicating the enhancement of QE radiation is attributed to the increase in hybridization when the defect energy level approaches the dark conduction band.

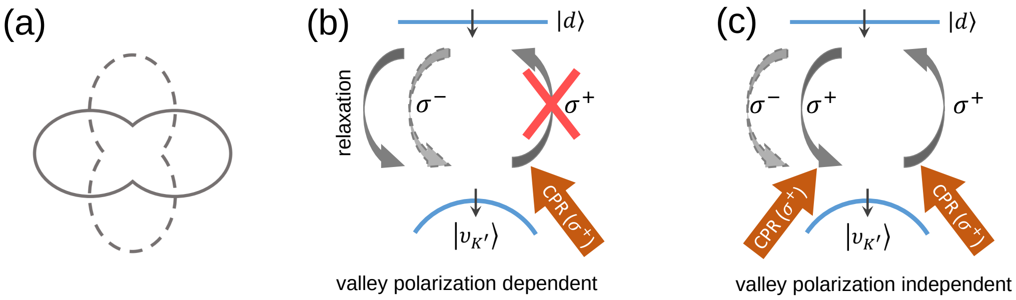

Supplementary Fig. 2. Polarization of output photon of QE coupled with CPR under different transition mechanisms. (a) Illustration of the emission of QE in a circular basis measurement at a large magnetic field, showing incomplete circularly polarized emission in each channel. (b) and (c) Illustration of the valley polarization dependent and independent transition processes respectively.

by various relaxation processes, such as phonon-assisted intervalley scattering, where the absorption process is not enhanced by CPR with cross-polarized, as shown in Fig. S2b. The presence of CPR may strengthen or weaken the relaxation process, leading to a further reduction or increase in the DCP, but not to a reversal of the DCP. For example, the scattering of valley excitons can be reduced by the microcavity [13, 14], but the valley polarization of a certain valley cannot be reversed. If it is not related to valley polarization, but the transition itself is not completely polarized. That means that the QE itself doesn't obey the selection rules strictly as the exciton angular momentum of QE is not a good quantum number. In this case, the transition of QE contains the components co-polarized and cross-polarized with CPR, then CPR can enhance not only the radiation, but also the co-polarized absorption process, resulting in a reversal of DCP.

SUPPLEMENTARY NOTE 2 - RESONANCE OF CHIRAL PLASMON LATTICES.

Figure S3 shows the reflection spectra of the chiral structures under different polarized excitations. Those structures are consistent with that in the main text. Here, we measured two same groups of chiral structures L1-L4 as a comparison. For the reflection spectra under linear excitation, we adjust the polarization of the incident light to be arranged along the x (P_x) and y (P_y) direction of lattice axes, as shown in the inset with SEM image. Both NPR and CPR can be seen under the cross-linearly polarized excitation, especially under P_x excitation. The energy of NPR mode shows no shift under different excitations, confirming that this resonance comes from the individual nanorods. The energy of CPR mode shows a slightly different for the two linearly polarized excitations in L1 and L4 because there's some adhesion between the unit cells along the x direction in these two structures. In L2 and L3, the cells are

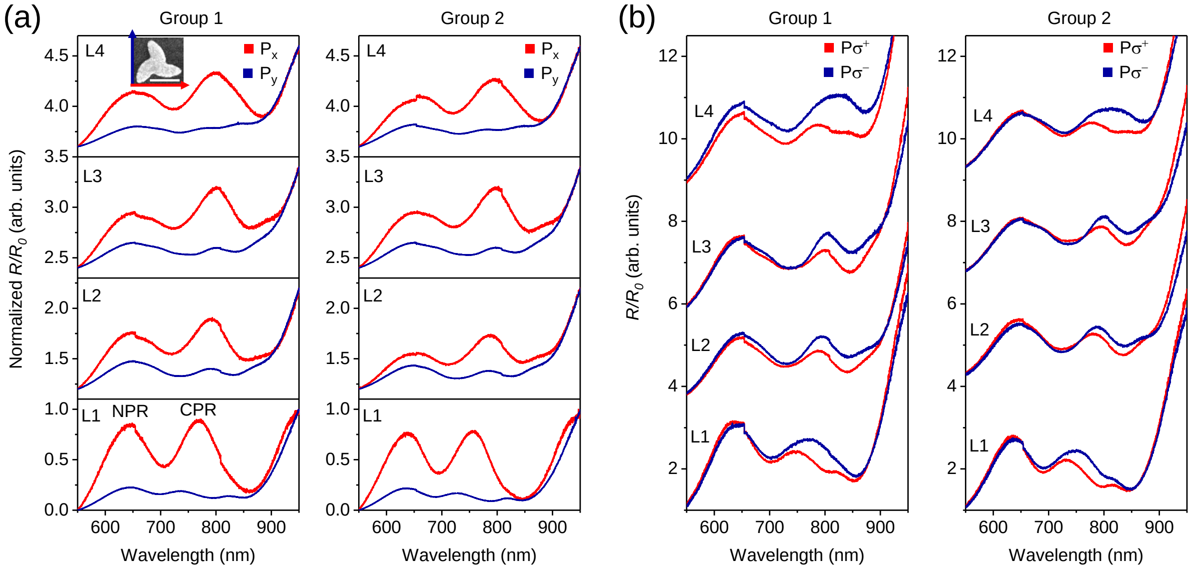

Supplementary Fig. 3. Reflection spectra from two groups of chiral structures under (a) linearly polarized excitation and (b) circularly polarized excitation. Scale bar of the SEM image is 250 nm. Here R and R_0 denote the reflection intensity taken from plasmonic nanostructures and the substrate, respectively.

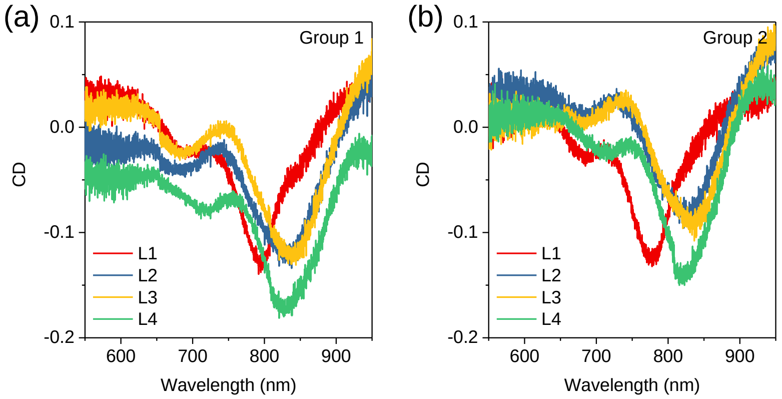

Supplementary Fig. 4. The CD spectra of two groups of lattices. (a) CD spectra of Group 1 lattices. (b) CD spectra of Group 2 lattices. Here, CD $= (I_{P_{\sigma+}} - I_{P_{\sigma-}})/(I_{P_{\sigma+}} + I_{P_{\sigma-}})$, where $I_{P_{\sigma+}}$ ($I_{P_{\sigma-}}$) denotes the intensity of the $P_{\sigma+}$ ($P_{\sigma-}$) polarized reflection spectra in Fig. S3b.

completely separated, so there is no obvious difference in energy under different polarized excitations.

Figure S3b and Figure S4 show the reflection spectra under cross-circularly polarized excitation and the corresponding circular dichroism (CD) spectra, respectively. Both of these spectra show similar results, indicating the robustness of our measurements. The CD value around the energy of CPR is relatively low (∼10-18%), as shown in Fig. S4. This is because the LCP and RCP components of CPR mode are evenly localized at different positions of the structure, which leads to the weak circular dichroism response on the overall spectrum. The adhesion between the unit cells in L1 and L4 results in the increase of the linewidth of CPR, as the above measured reflection spectra and corresponding simulations in Fig. S5a show. But it has a slight influence on the properties of chirality of the CPR mode, as the calculated optical chirality enhancement in Fig. S5b shows.

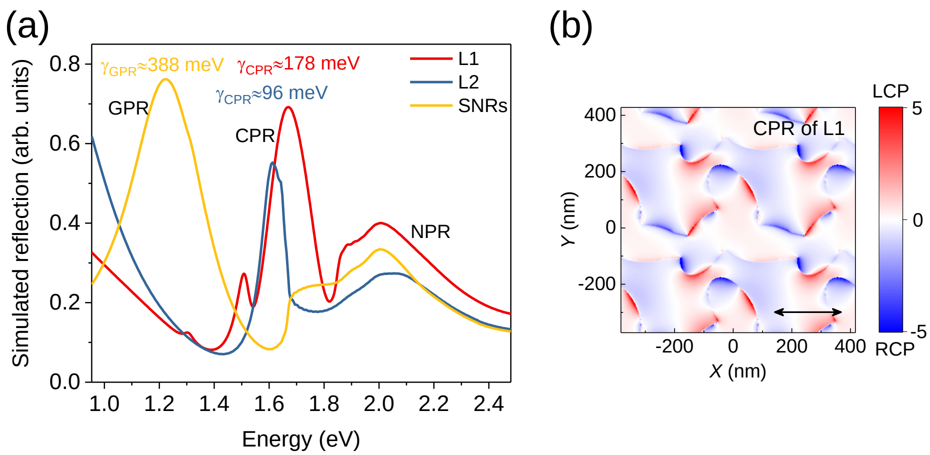

Supplementary Fig. 5. Numerical simulation of L1, L2 and separated nanorods (SNRs). (a) The simulated reflection spectra of L1, L2 and SNRs, where the simulation results of L1 and L2 agree with the measured ones in Fig. 2c in the main text. The linewidth of the resonance of gap plasmon is several times larger than that of the chiral plasmon, indicating a lower quality factor of GPR. (b) The calculated optical chirality enhancement of CPR of L1, shows a similar distribution with L2 (Fig. 1g in the main text). The black arrow indicates the polarization direction of incident light.

SUPPLEMENTARY NOTE 3 - THE g-FACTORS OF QES IN WSE_2 MONOLAYER.

Figure S6 shows the magnetic field dependence of PL in the Voigt geometry. As we can see, there is no observable peak shift for all QEs in the whole range of B variations, indicating a g-factor $= 0$ to an in-plane magnetic field. This means the spin degree of freedom is frozen out and does not contribute to the B-induced splitting [15]. When applied with the out-of-plane magnetic field, we observe large g-factors similar to the previous literatures [16–18], as shown in Fig. S7. Owning to the hybridization with the lowest dark conduction band, only the low-energy peak is visible in the PL, especially at higher B. Thus, here we fit the g-factors of the QEs by assuming a symmetric energy splitting at the non-zero magnetic field [19]. That is

$$E_L = E_0 - \frac{1}{2}\sqrt{{\delta_0}^2 + (g\mu_B B)^2} \tag{19}$$

$$E_H = E_0 + \frac{1}{2}\sqrt{{\delta_0}^2 + (g\mu_B B)^2} \tag{20}$$

$$\Delta(E) = E_H - E_L = \sqrt{{\delta_0}^2 + (g\mu_B B)^2} \tag{21}$$

Here, E_0 is the energy of the quantum emitter without electron-hole exchange interaction, δ_0 is the zero-field fine structure splitting, μ_B is the Bohr magneton, g is the Land$\acute{e}$ factor, E_L and E_H represent the energies of low energy and high energy peaks, respectively. Therefore, we can fit the g-factors of the QEs by only one energy peak branch, such as the visible low energy peak as shown in Fig. 3 in the main text. The values of the g-factors range from 10 to 12, and the average value for 13 QEs is 11.03 ± 0.44.

The extreme anisotropic response of QEs in TMDs layers to an in-plane versus out-of-plane magnetic field is similar to that of two-dimensional valley exciton. Consequently, previous reports believe that the QEs in the layer inherit the valley physics and share the band structure of two-dimensional excitons [20]. However, this assumption can not give a reasonable explanation on the g-factors in an out-of-plane magnetic field. The value of QEs around 10-12 is more than twice as that of the two-dimensional exciton (around 4.4). The large g-factor can be well explained with considering the QEs as intervalley defect excitons. Due to the lack of conduction band participation in the transition of intervalley defect exciton, the orbital magnetic moment of valley can provide a net valence band shift when applying an out-of-plane magnetic field. Considering the spin magnetic moment does not contribute to the splitting, the net magnetic moment of the QEs consists of two parts: $M = \alpha\tau\mu_B + m\tau\mu_B$, where $\alpha\tau\mu_B$ is the valley magnetic moment and $m\tau\mu_B$ is the magnetic moment from the contribution of the $d-$orbitals of the transition metal with m=2. Here, $\tau = \pm 1$ is the index for valence bands at K and $K^{'}$ Valleys. The valley g-factor α for the valence band is ~ 3.5 when we take $g = 11.03$.

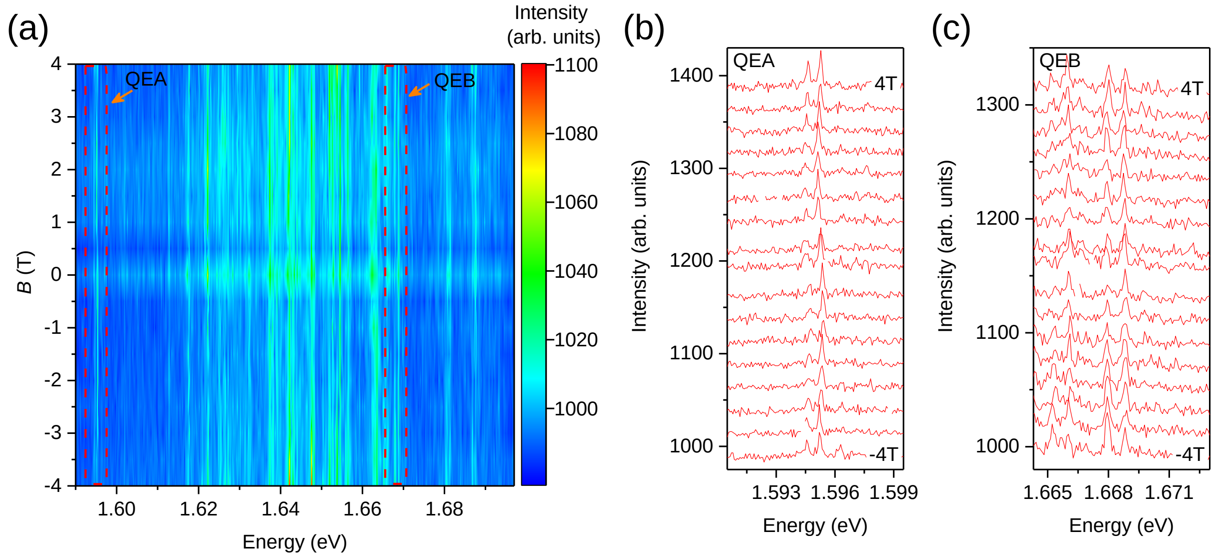

Supplementary Fig. 6. (a) Magnetic field dependence of PL mapping from QEs at L1 as shown in the main text in the Voigt geometry (The magnetic field is parallel to monolayer). There is no observable energy shift as B changes from -4T to 4T for all QEs. (b) and (c) The Magnetic field dependent PL of two selected typical quantum emitters QEA and QEB, as marked by the dashed red box in (a). The spectra are shifted for clarity.

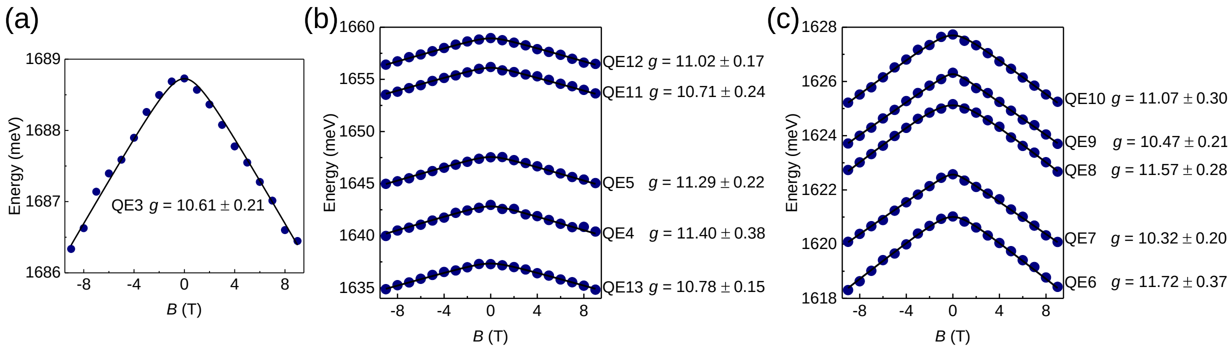

Supplementary Fig. 7. The g-factors of QEs in the main text. (a) (b) and (c) Fitted g-factors of QE3-QE13. All QEs were measured from L1 in the Faraday geometry, where QE3-QE10 correspond to labels marked in the main text.

SUPPLEMENTARY NOTE 4 - CIRCULAR PHOTOLUMINESCENCE POLARIZATION OF THE EMISSION FROM WSE_2 MONOLAYER ON CHIRAL PLASMON.

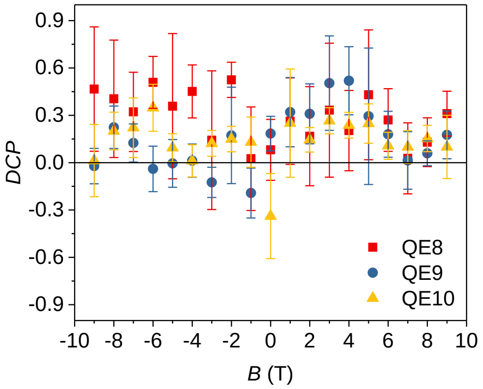

Supplementary Fig. 8. The DCP of QE8-10 calculated from the spectra in Fig. 3i in the main text. The values show a weaker σ^+ polarized emission comparing to QE7, indicating those QEs are located in the region where the intensity of chiral plasmon field is relatively weak. The source of error bars is the standard error of QE intensity in peak fitting.

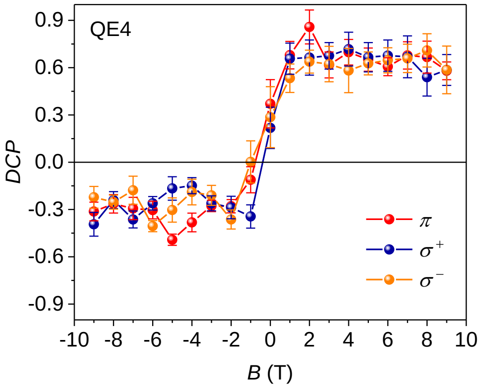

Supplementary Fig. 9. The DCP of QE4 under linearly (π), σ^+ and σ^- polarized excitations. There is no observable difference of DCP under σ^+ and σ^- polarized excitations. The source of error bars is the standard error of QE intensity in peak fitting.

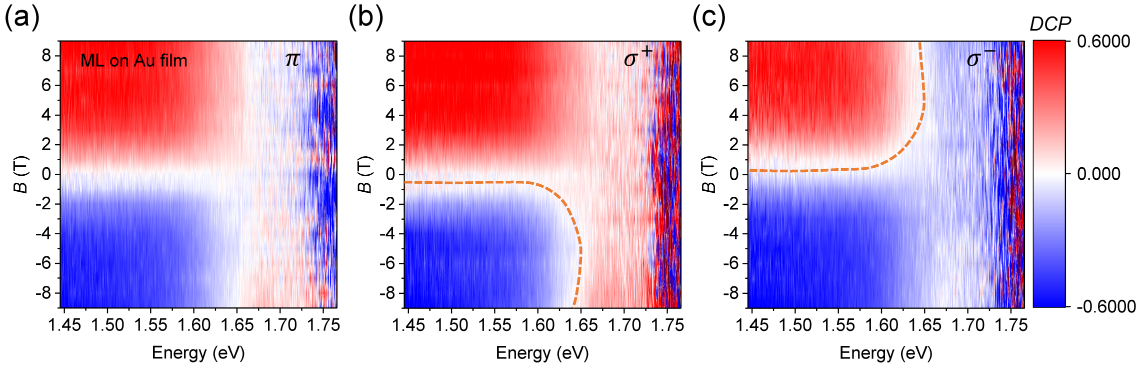

Supplementary Fig. 10. The DCP of WSe_2 monolayer on Au film under excitation with (a) π, (b) σ^+ and (c) σ^- polarized light. The PL with energy lower than 1.65 eV comes from the emission of defect excitons. Under circularly polarized excitation, the PL with energy higher than 1.65 eV shows an apparent valley polarization, which stems from the valley polarization of neutral exciton and trion resonance i.e. transition between the conduction band and the valence band.

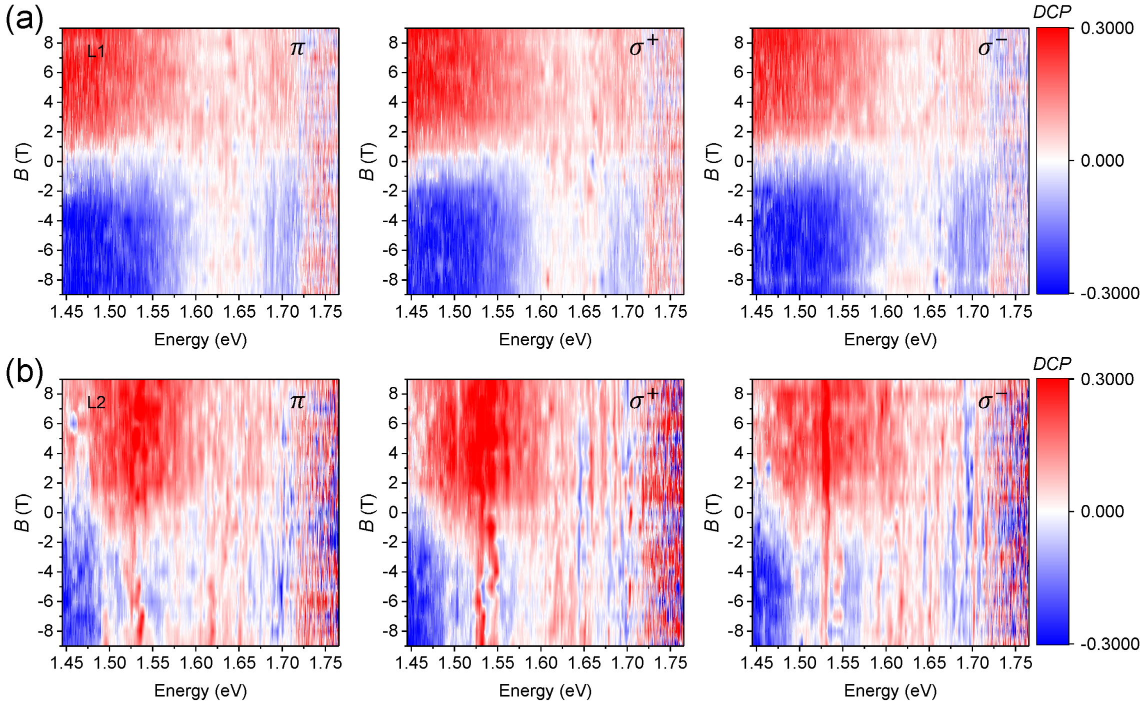

Supplementary Fig. 11. The DCP of WSe_2 monolayer on (a) L1 and (b) L2 under π, σ^+ and σ^- excitations. There is no observable difference in spectra under different polarized excitations, confirming the absence of the valley polarization for those localized defect excitons.

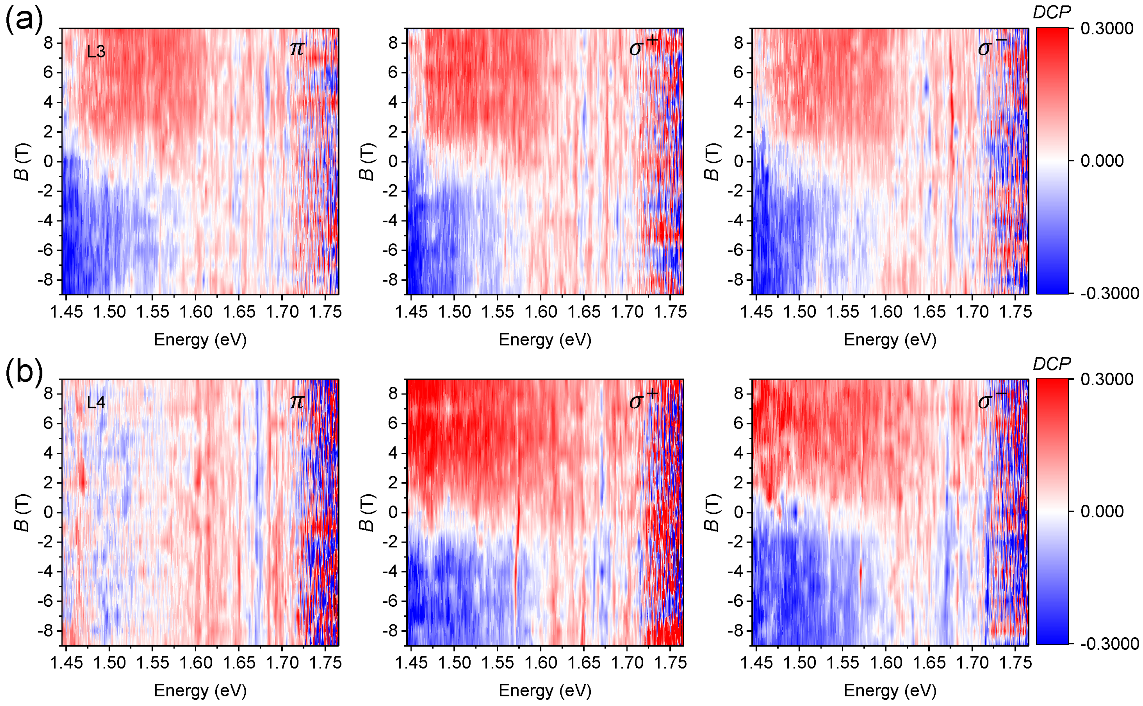

Supplementary Fig. 12. The DCP of WSe_2 monolayer on (a) L3 and (b) L4 under π, σ^+ and σ^- excitations. The results are similar to that in Fig. S11. At L4, some differences between the results of linearly polarized excitation and circularly polarized excitation may be due to the measurements at different positions (but still at L4).

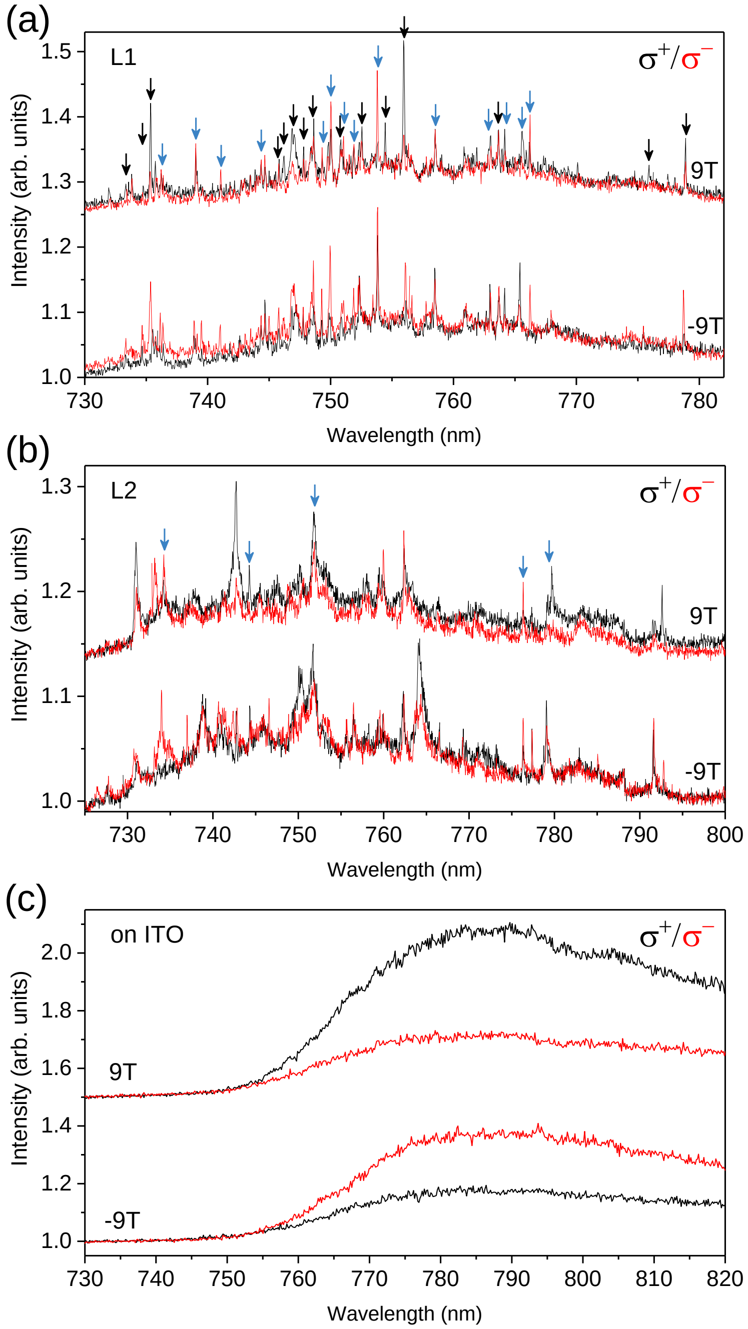

Supplementary Fig. 13. Statistics on the QEs coupled to CPR. The spectra show the polarization-resolved PL from QEs (a) at L1, (b) at L2 and (c) on ITO substrate, respectively. The results were measured under -9 T and 9 T magnetic field and were detected in the circular basis (black lines: σ^+ configuration; red lines: σ^- configuration). The black and blue arrows represent the QEs that are not coupled to CPR and QEs that are coupled to CPR, respectively. To count the number of coupled and uncoupled QEs, we only show the results of 9 T and -9 T. Since only low energy peaks occur at high magnetic fields, each narrow linewidth single peak represents the radiation of a QE. As shown in Fig. S13a, we observed a large number of narrow linewidth emissions at L1 due to the effective matching of defect energies and CPR mode. We performed statistics on the coupled QEs with anomalous behavior, that is, QEs with the same helicities under opposite magnetic fields, as shown by the blue arrows. We find that among 29 QEs, 14 QEs have anomalous behavior, indicating a high proportion of QEs coupled to CPR. This high proportion is highly correlated with the fact that QEs are more likely to be generated at the edge and that the chiral field is mainly distributed at the edge of the nanostructure. QEs coupled to CPR can also be found at L2 as shown in Fig. S13b, though the number of individual QEs with narrow linewidths is much lower than at L1 due to poor resonance with the CPR mode. But at both L1 and L2, the results on the chiral nanostructure are in stark contrast to those on the substrate.

SUPPLEMENTARY NOTE 5 - THE POWER DEPENDENT PL AND LINEAR POLARIZATION-DEPENDENT PL OF DEFECTS FROM WSE_2 LAYERS.

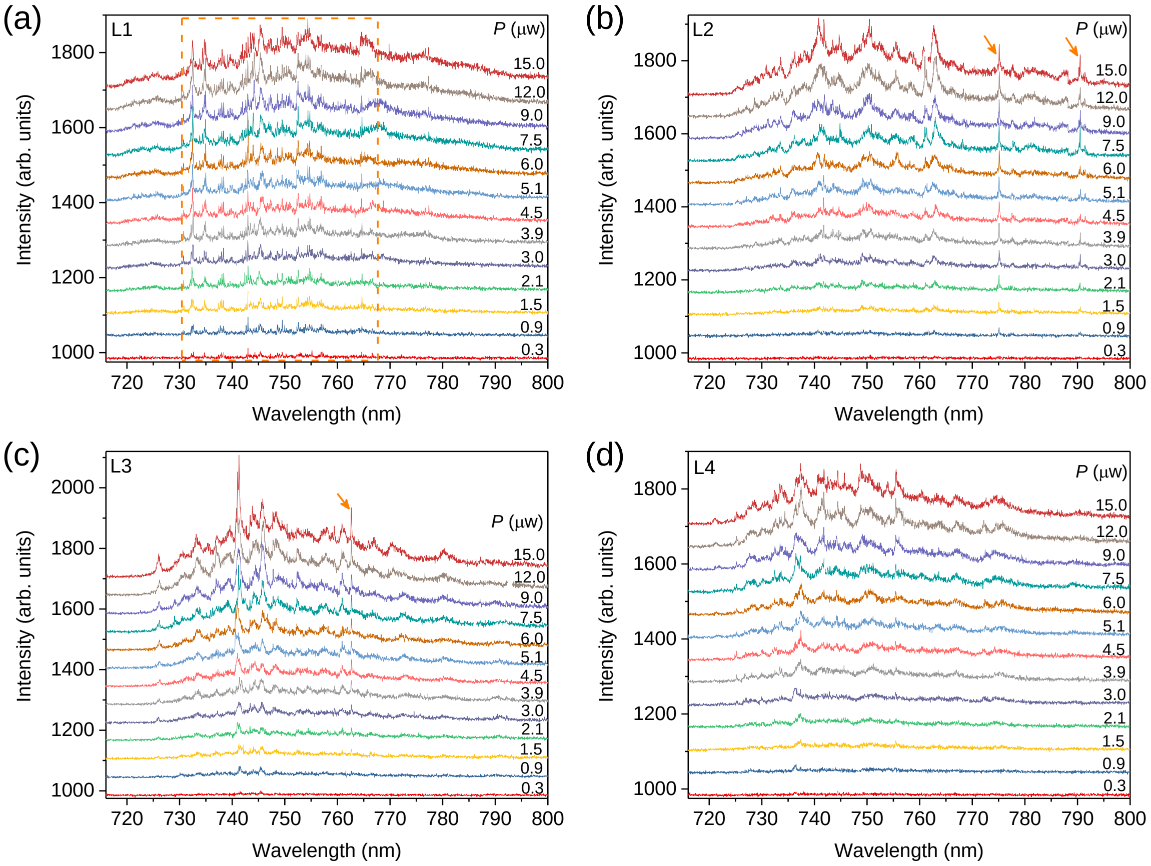

Supplementary Fig. 14. Power (P) dependent PL spectra of the sample in the main text. (a) (b) (c) and (d) The PL spectra of the monolayer on structures L1-L4. Different from L1 with the emission of massive isolated QEs (orange dashed box), only several narrow peaks can be seen in L2-L4, as marked with orange arrows.

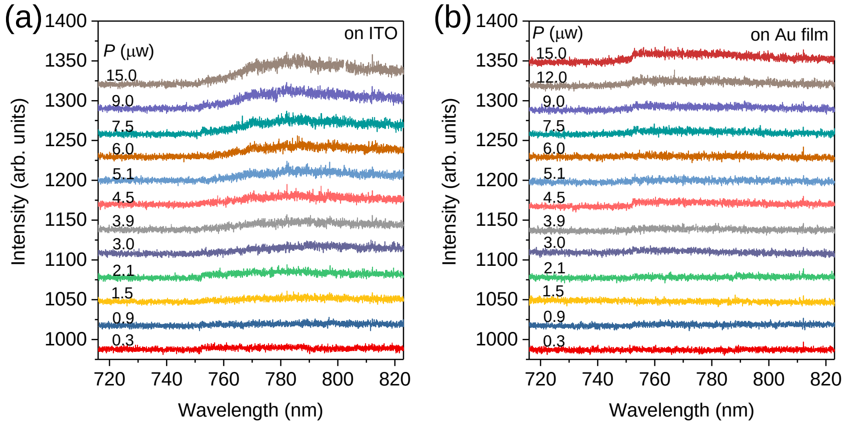

Supplementary Fig. 15. Power dependent PL of the monolayer on (a) ITO substrate and (b) Au film. No individual narrow peak can be resolved.

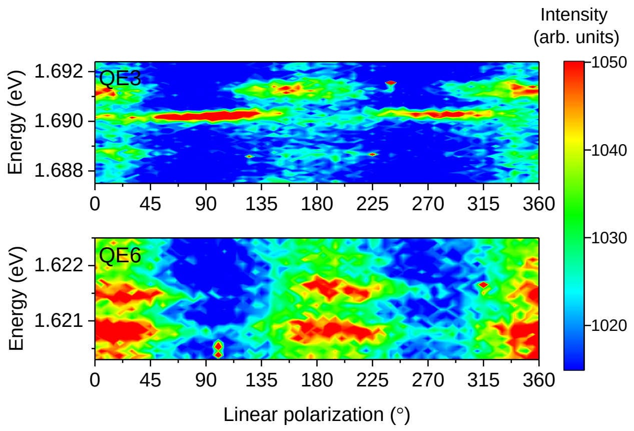

Supplementary Fig. 16. PL-intensity plot of QE3 (top) and QE6 (bottom) detected in linear basis under zero magnetic field. A pair of cross-linearly polarized spectral doublet is observed for the uncoupled QE3 due to the electron-hole exchange interaction as shown in the top plot, consistent with those reported in the literatures. For the QE coupled to CPR, we do not observe this cross-linearly polarized spectral doublet similar to QE3. Instead, we observe a pair of elliptically polarized spectral doublet with the same polarization properties, such as the measurement results in QE6, as shown in the lower plot. This indicates that both emission modes of fine-structure split doublet at zero magnetic field are coupled to chiral plasmon, and that their polarization properties are strongly modulated by the chiral field. For the elliptically polarized light emitted by the coupled QE, the degree of circular polarization of the output photon can be further improved by the chiral plasmon field with higher optical chirality.

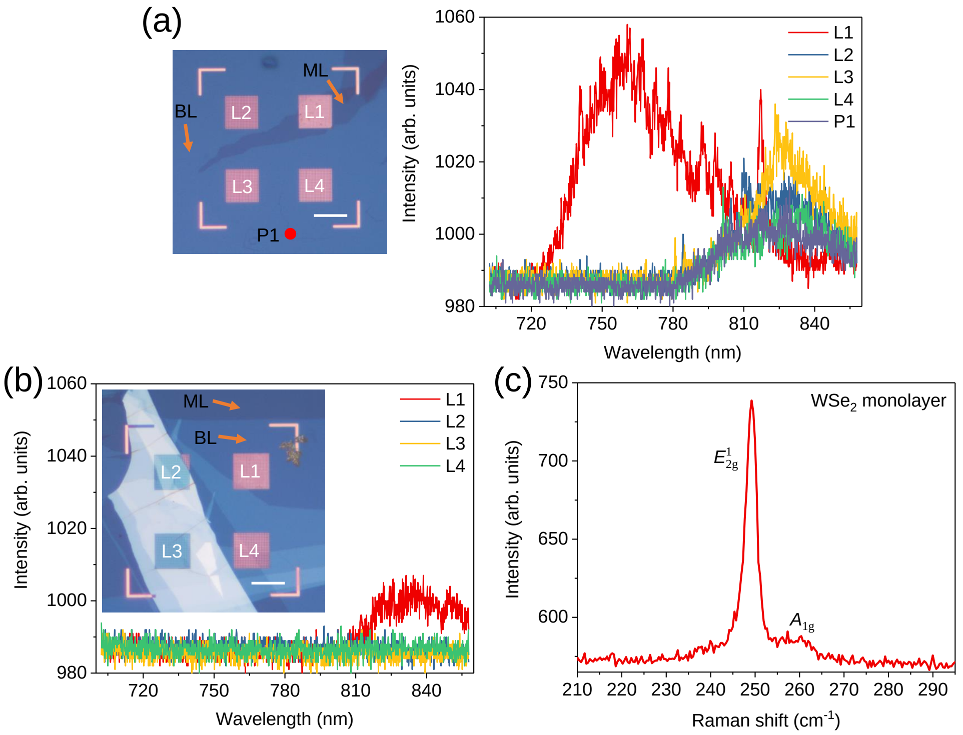

Supplementary Fig. 17. PL spectra of other samples. (a) and (b) The measured PL spectra of the samples covered with WSe_2 monolayer (ML), bilayer (BL) and multi-layers at 4.2 K. The bilayer exhibits a weak PL signal with a large red shift in energy comparing with monolayer. Scale bar, 10 μm. As for multilayer, no observable emission can be seen in spectra. This significant spectral difference between monolayer and bilayer or multilayer makes it easy to identify monolayer. (c) The Raman spectrum of WSe_2 monolayer at room temperature with in-plane phonon mode (E^1_{2g}) and out-of-plane phonon mode (A_{1g}), which is consistent with the literature [21].

SUPPLEMENTARY NOTE 6 - OPTICAL CHARACTERIZATIONS OF THE INTERACTION BETWEEN CHIRAL PLASMON AND $MOSE_2$ MONOLAYER.

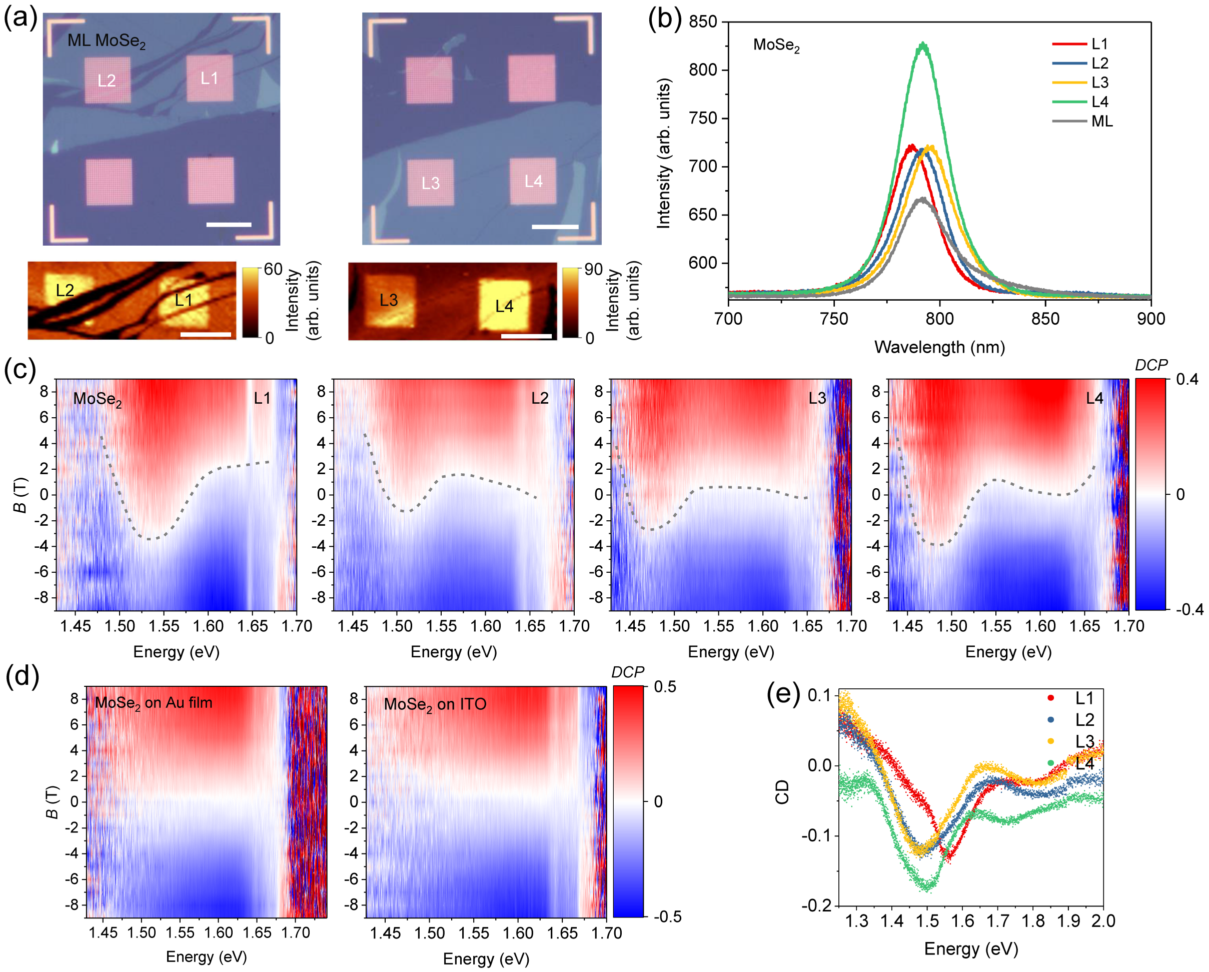

Supplementary Fig. 18. Optical characterizations of the interaction between the chiral plasmon and the $MoSe_2$ monolayer. (a) Optical microscope images of $MoSe_2$ monolayer covered on the plasmonic lattices L1-L4. Bottom: Corresponding PL mapping at 792 nm. Scale bar, 10 μm. (b) The PL spectra of the hybrid structures. The measurements in (a) and (b) were taken at room temperature. The emission peaks of excitons are modified by the chiral plasmon resonances compared to the measured reflection spectra as shown in Fig. 2c in the main text. (c) and (d) Measured magnetic field dependent DCP of the $MoSe_2$ monolayer covered on plasmonic lattices L1-L4 and substrates, respectively. The corresponding measurements were performed with an excitation by a linearly polarized light at 4.2 K. The gray dashed lines in (c) mark the points where DCP equals to zero. (e) The measured CD spectra of plasmonic lattices L1-L4. By comparing the gray dashed lines in (c) with the CD spectra in (e), we can find that the circularly polarized emissions of the massive defects can be modified by the whole chiral plasmon resonance in the far field at lower magnetic field. When the applied magnetic field is higher than 2-4 T, the DCP at the lattices changes back to the same results as that on ITO and Au film substrates, which is apparently different from that in WSe_2 as shown in Fig. 4d in the main text. The defect excitons in $MoSe_2$ monolayer are intravalley defect excitons, unlike in WSe_2, the defect states in $MoSe_2$ do not need to undergo cross-valley hybridization to produce a strong photon emission, thus preserve the valley-dependent selection rules. This anomalous circularly polarized emission at low magnetic field can be attributed to the strong electron-hole exchange interaction and the weak thermal relaxation of high energy peaks. As the magnetic field increases, the electron-hole exchange in the defect excitons is overcome and the emissions become circularly polarized. Meanwhile, with the increase of the magnetic field, the intensity of high energy peaks gradually weakens due to the strengthening of thermal relaxation. Due to the valley-dependent selection rules, the circularly polarized emission of low energy peaks will no longer be modified by the chiral plasmon field. Therefore, the circularly polarized emissions will not have a reversal of DCP similar to that in WSe_2 monolayer.

SUPPLEMENTARY REFERENCES

[1] D. G. Angelakis, Quantum simulations with photons and polaritons, Quantum Science and Technology (Springer, 2017) , 134 (2017).

[2] D. Manzano, A short introduction to the lindblad master equation, AIP Advances **10**, 025106 (2020).

[3] C.-N. Yang, Selection rules for the dematerialization of a particle into two photons, Physical Review **77**, 242 (1950).

[4] G. Wysin, Quantization of the free electromagnetic field: Photons and operators, Department of Physics, Kansas State University (2011).

[5] A. Trügler and U. Hohenester, Strong coupling between a metallic nanoparticle and a single molecule, Physical Review B **77**, 115403 (2008).

[6] J. R. Johansson, P. D. Nation, and F. Nori, QuTiP: An open-source python framework for the dynamics of open quantum systems, Computer Physics Communications **183**, 1760 (2012).

[7] J. R. Johansson, P. D. Nation, and F. Nori, QuTiP: An open-source python framework for the dynamics of open quantum systems, Computer Physics Communications **184**, 1234 (2013).

[8] D. Yang, X. Fan, F. Zhang, Y. Hu, and Z. Luo, Electronic and magnetic properties of defected monolayer WSe_2 with vacancies, Nanoscale Research Letters **14**, 192 (2019).

[9] E. Liu, J. van Baren, C.-T. Liang, T. Taniguchi, K. Watanabe, N. M. Gabor, Y.-C. Chang, and C. H. Lui, Multipath optical recombination of intervalley dark excitons and trions in monolayer WSe_2, Physical Review Letters **124**, 196802 (2020).

[10] P. Peng, Y.-C. Liu, D. Xu, Q.-T. Cao, G. Lu, Q. Gong, and Y.-F. Xiao, Enhancing coherent light-matter interactions through microcavity-engineered plasmonic resonances, Physical Review Letters **119**, 233901 (2017).

[11] E. Waks and D. Sridharan, Cavity QED treatment of interactions between a metal nanoparticle and a dipole emitter, Physical Review A **82**, 043845 (2010).

[12] R. A. Matula, Electrical resistivity of copper, gold, palladium, and silver, Journal of Physical and Chemical Reference Data **8**, 1147 (1979).

[13] Y.-J. Chen, J. D. Cain, T. K. Stanev, V. P. Dravid, and N. P. Stern, Valley-polarized exciton–polaritons in a monolayer semiconductor, Nature Photonics **11**, 431 (2017).

[14] S. Dufferwiel, T. P. Lyons, D. D. Solnyshkov, A. A. Trichet, F. Withers, S. Schwarz, G. Malpuech, J. M. Smith, K. S. Novoselov, M. S. Skolnick, *et al.*, Valley-addressable polaritons in atomically thin semiconductors, Nature Photonics **11**, 497 (2017).

[15] A. Srivastava, M. Sidler, A. V. Allain, D. S. Lembke, A. Kis, and A. Imamoğlu, Valley zeeman effect in elementary optical excitations of monolayer WSe_2, Nature Physics **11**, 141 (2015).

[16] A. Srivastava, M. Sidler, A. V. Allain, D. S. Lembke, A. Kis, and A. Imamoğlu, Optically active quantum dots in monolayer WSe_2, Nature Nanotechnology **10**, 491 (2015).

[17] M. Koperski, K. Nogajewski, A. Arora, V. Cherkez, P. Mallet, J.-Y. Veuillen, J. Marcus, P. Kossacki, and M. Potemski, Single photon emitters in exfoliated WSe_2 structures, Nature Nanotechnology **10**, 503 (2015).

[18] Y.-M. He, G. Clark, J. R. Schaibley, Y. He, M.-C. Chen, Y.-J. Wei, X. Ding, Q. Zhang, W. Yao, X. Xu, C.-Y. Lu, and J.-W. Pan, Single quantum emitters in monolayer semiconductors, Nature Nanotechnology **10**, 497 (2015).

[19] Q. Wang, J. Maisch, F. Tang, D. Zhao, S. Yang, R. Joos, S. L. Portalupi, P. Michler, and J. H. Smet, Highly polarized single photons from strain-induced quasi-1D localized excitons in WSe_2, Nano Letters **21**, 7175 (2021).

[20] X. Lu, X. Chen, S. Dubey, Q. Yao, W. Li, X. Wang, Q. Xiong, and A. Srivastava, Optical initialization of a single spin-valley in charged WSe_2 quantum dots, Nature Nanotechnology **14**, 426 (2019).

[21] H. Zeng, G.-B. Liu, J. Dai, Y. Yan, B. Zhu, R. He, L. Xie, S. Xu, X. Chen, and W. Yao, Optical signature of symmetry variations and spin-valley coupling in atomically thin tungsten dichalcogenides, Scientific Reports **3**, 1608 (2013).
